# The complete ITS2 barcoding region for *Strongylus vulgaris* and *Strongylus edentatus*

**DOI:** 10.1007/s11259-022-10067-w

**Published:** 2023-01-04

**Authors:** Peter Halvarsson, Eva Tydén

**Affiliations:** Department of Biomedical Sciences and Veterinary Public Health, Swedish University of Agricultural Sciences, PO Box 7036, 750 05 Uppsala, Sweden

**Keywords:** Metabarcoding, Nemabiome, Internal transcribed spacer, Species determination, Parasite, Strongyles

## Abstract

Gastrointestinal nematode parasites are of major concern for horses, where *Strongylus vulgaris* is considered the most pathogenic among the *Strongylus* species. Diagnosis of *S. vulgaris* infections can be determined with next generation sequencing techniques, which are inherently dependent on reference sequences. The best marker for parasitic nematodes is internal transcribed spacer 2 (ITS2) and we provide the first complete ITS2 sequences from five morphologically identified *S. vulgaris* and additional sequences from two *S. edentatus*. These sequences have high similarity to already published partial sequences and amplicon sequence variants (ASV) based on next generation sequencing (NGS). The ITS2 sequences from *S. vulgaris* matched available partial ITS2 sequences and the full ASVs, whereas the *S. edentatus* sequence matched another complete sequence. We also compare Sanger sequencing and NGS methods and conclude that the ITS2 variation is better represented with NGS methods. Based on this, we recommend that further sequencing of morphologically identified specimens of various species should be performed with NGS cover the intraspecific variation in the ITS2.

**Supplementary Information:**

The online version contains supplementary material available at 10.1007/s11259-022-10067-w.

Gastrointestinal nematode (GIN) parasite infections are a major concern for equine industry as it affects both horse health and welfare. Grazing horses can be infected by over 50 different species of GINs (Bellaw and Nielsen 2020). Infection rates can be up to 100% for small strongyles (Morariu et al. 2016) but for *Strongylus* spp. the prevalence is lower (Campbell et al. 1995; Hung et al. 1996; Poissant et al. 2021). In the 1970-ies, prior to general anthelmintic treatment, *S. vulgaris* had a prevalence of 80–100% (Slocombe and McCraw 1973; Tolliver et al. 1987; Nielsen et al. 2012). Intense use of anthelmintics and the long life cycle reduced the prevalence to 5% in the 1990-ties (Craven et al. 1998; Studzińska et al. 2012).

Infections with *Strongylus vulgaris* are considered to be the major parasite nematode causing disease and death in horses (Gonzales-Viera et al. 2019), and other *Strongylus* species, such as *S. edentatus* affect the horse to a lesser extent (McCraw and Slocombe 1974). The life cycle of strongyle species is direct where eggs, which are passed out with feces, develop into larvae on the pasture. Strongyles exhibit three sequential larval stages, first (L1), second (L2), and third (L3), where L3 is the infective stage. Thereafter the life-cycle is somewhat different between *S. vulgaris* and *S. edentatus.* The life-cycle of *S. vulgaris* includes migration of larvae to the cranial mesenteric arteries where the larvae stay for several months and develop to L4 and subsequently to L5 before migrating downstream to enter the lumen as adults in the large intestines (Duncan and Pirie 1972).The pathogenicity of *S. vulgaris* is related to the migration of larvae in the mesenteric arteries where arteritis, hemostatic changes and thrombosis may cause thrombo-embolic colic with non-strangulating intestinal infarctions (NSII) (Pihl et al. 2018; Hedberg-Alm et al. 2022). Contrasting *S. vulgaris*, *S. edentatus* L4 larvae migrates from colon to the liver, and then return to the intestine where they develop to adults (McCraw and Slocombe 1974). Therefore, it is important to diagnose *Strongylus* infections.

Diagnosing strongyle eggs from fecal matter using microscopy is not difficult but the morphology of the eggs does not allow to differentiate between migratory and non-migratory strongyles, and between the different genera and species. Traditionally, a larval culture and microscopy are needed to identify the large strongyles (Roeber et al. 2013). Recently, a way of overcoming the species identification problem have been implemented by taking advantage of metabarcoding using next generation sequencing (NGS) technologies. In metabarcoding, multiple samples are metabarcoded with unique sequence tags, multiplexed and sequences which enables identification of all parasitic nematode species infecting the host at the same time, also known as “nemabiome” (Avramenko et al. 2015). DNA for this method can be extracted directly from fecal matter or from larval cultures (Avramenko et al. 2015; Poissant et al. 2021; Halvarsson and Höglund 2021). Internal transcribed spacer region 2 (ITS2) is the standard choice for metabarcoding of GINs (Avramenko et al. 2015). The ribosomal DNA region where ITS2 is located is a multicopy tandem repeated array, thus many copies of the ITS2 can be obtained from the same sample and each of the copies can differ slightly in their sequence yielding within-individual variation (Marek et al. 2010). The different species can be identified by the ITS2 sequence, however the method is highly dependent on available ITS2 reference sequences in public databases (Workentine et al. 2020). There are still ITS2 sequences missing for various species. One example is *S. vulgaris*, where only partial ITS2 sequences are available in NCBI GenBank.

In this study we Sanger sequenced the ITS2 region of *S. vulgaris* and *S. edentatus*. Five *S. vulgaris* L4 were collected from the mesenteric artery from a 10-year-old Arabian horse. Two pre-adult *S. edentatus* were collected from nodules of peritoneal wall on the right abdominal region of the body of a 17-year-old Tinker. Both horses were euthanized at the University Animal Hospital and autopsied at the Swedish Agricultural Sciences, Sweden. These sequences were compared to available ITS2 data from literature, and they can be used for future NGS metabarcoding studies.

DNA was extracted from each *Strongylus* specimen after they were fragmented using the Nucleospin® DNA tissue kit (Macherey–Nagel, Düren, Germany) following the manufacturer’s protocol. The complete ITS2 region was amplified in 10 µl PCR reactions with the primer pair NC1/NC2 described in Gasser et al. (1993), using PCRBIO HS VeriFi™ Mix (PCR biosystems, London, UK) according to the manufacturer’s standard instructions. 1 µl DNA (0.2–10 ng measured with NanoDrop) was used as template. PCR products were sent to Macrogen Europe for post-PCR cleanup and Sanger sequencing in both directions with the same primers as for the PCR amplification.

The obtained forward and reverse DNA sequences were assembled using the software CodonCodeAligner v10.0.2. The assembled sequences were 275 bp for *S. vulgaris* and 293 bp for *S. edentatus* after removing primer sequences. These were compared to NCBI GenBank records to find the best matches. The sequences matching *S. vulgaris* and *S. edentatus* were downloaded from NCBI GenBank together with sequences from *S. equinus* and *S. asini*. In addition to the NCBI GenBank sequences, we ran the pipeline script published by Poissant et al. (2021) where horse nemabiomes were studied. We chose to include the 11 most common *S. vulgaris* sequences from the pipeline based on how many sequences were found in the dataset. These 11amplicon sequence variant (ASV) sequences each had > 1000 reads for *S. vulgaris, S. edentatus and E. equinus*. These sequences were combined with the sequenced specimens in this study (see online resource 1, tab. S1 for details). A subset containing only complete sequences and scaffolds was also created. Sequences for both datasets were uploaded to the website phylogeny.fr. ‘Advanced’ mode was chosen for phylogeny analysis, where the sequences were aligned with MUSCLE in ‘full mode’, all other settings at default values to create maximum likelihood phylogenetic trees (Dereeper et al. 2008). The obtained trees were loaded to the R v4.2.1 (R Core Team 2022) environment with Treeio v1.20.2 (Wang et al. 2020) and visualized using package ggtree v3.4.4 (Yu et al. 2017). The alignment in Fig. 1 was created using BIOEDIT v7.2.6.1 (Hall 1999).

**Fig. 1:**
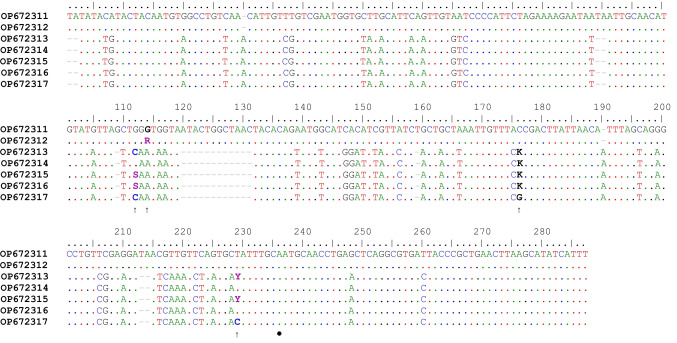
Alignment of complete ITS2 (bases 1–236 in the alignment) sequences from *Strongylus edentatus* (two top sequences) and *S. vulgaris* (five bottom sequences) amplified with the primers NC1 and NC2. The arrows under the alignment indicate polymorphic sites, and the dot indicate the end of the ITS2

All five ITS2 sequences obtained from *S. vulgaris* were unique and they had unique combinations of intraspecific base pair positions, and one specimen of *S. edentatus* had a single intraspecific site in the ITS2 region sequence (Fig. 1). These polymorphic sites could be a result of the multicopy tandem repeated array structure of the ribosomal DNA (rDNA). Nevertheless, the sequences showed high to a perfect match (98.6–100%) to the partial sequences available on NCBI GenBank (Online resource 1, tab S2). These seven sequences have been uploaded to NCBI GenBank under accession numbers OP672311—OP672317.

Sequences from the five *S. vulgaris* in this study clustered together with the other sequences in a monophyletic clade in the phylogenetic tree based on partial ITS2 sequences. The clade for *S. edentatus* and *S. equinus* was not well resolved. Due to the 77 bp short-length sequences, the tree is poorly resolved, but still places *S. asini* closer to *S. edentatus* and *S. equinus* than to *S. vulgaris* (Fig. 2A).

**Fig. 2:**
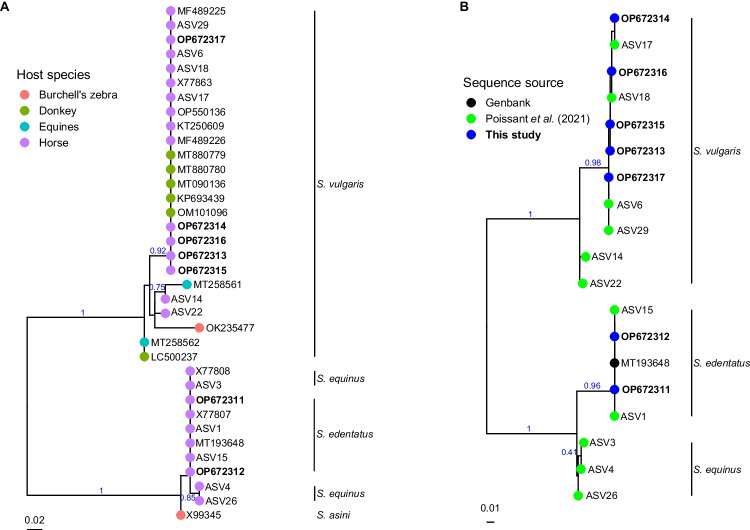
Phylogenies based on **A**) the partial sequences of the ITS2 region for the four *Strongylus* species (based on 77 bp) and in 2**B**) the available complete sequences. The ASV with the lowest number is the most common ASV in the Poissant et al. (2021) paper, and the higher the ASV number, the rarer the sequence. The phylogenies are based on maximum likelihood. Accession numbers in **bold** indicate the sequences from this study

The complete-ITS2-tree formed a well resolved clade for each of the three species, where there are two ASV sequences placed more basal in the *S. vulgaris* clade (Fig. 2B). The tree in Fig. 2B is based on 263 base pairs. Five *S. vulgaris* samples from donkeys (China, Iran and Egypt) clustered in the center of the *S. vulgaris* branch in Fig. 2A, however, these results should be interpreted with caution due to the short sequence length that the tree is based on. Otherwise, the host species were scattered among the sequences in both phylogenetic trees.

Metabarcoding of GIN parasite species relies on ITS2 sequences provided from morphologically identified specimens and making these sequences available are indispensable. To our knowledge, we are providing the first complete ITS2 sequences from *S. vulgaris* and additional for *S. edentatus*. These sequences are indispensable as reference sequences for metabarcoding projects, molecular identification, and diagnosis. These sequences from our samples had a high identity to the partial sequences available on NCBI GenBank, as well as the complete ASV sequences from data published in Poissant et al. (2021) (Fig. 2B). An advantage of Sanger sequencing is that it is cheap and readily available for diagnosing an infection, and between-individual variation can be detected for the most common variant(s). At the same time, a drawback with Sanger sequencing is that when all ITS2 variants in the multicopy tandem repeated array are amplified, the signature of these can be added as polymorphic sites, but the underlying variants cannot be detangled. Our data demonstrate the large variation in the ITS2, where five individuals showed unique combinations of polymorphic sites. NGS contrasts Sanger sequencing in this aspect, where different variants can be sequenced separately and the corresponding ASVs yields additional information, illustrated by the ASVs at the deeper nodes within the *S. vulgaris* clade (Fig. 2B). This is seen just by adding the most common ASVs. Overall, the results support the notion that NGS provide a better resolution in number of ITS2 haplotypes which has been demonstrated for fungi (Estensmo et al. 2021), wildlife (Beaumelle et al. 2021; Halvarsson et al. 2022), horses (Poissant et al. 2021) and sheep (Avramenko et al. 2015; Halvarsson and Höglund 2021). Despite the large intra-specific variation in the ITS2 region, it is still easy to distinguish between species because in addition to sequence variation, the ITS2 region varies in length depending on species (Poissant et al. 2021; Halvarsson and Höglund 2021).

To conclude, sequencing the whole ITS2 region of morphologically determined species, not previously in public databases, are invaluable for GINs studies. Even a single sequence from Sanger sequencing is valuable, but to capture the intraspecific variation, NGS using metabarcoded samples is recommended.

## Supplementary Information

Below is the link to the electronic supplementary material.

Supplementary file1 (PDF 292 KB) Online resource 1, contains a table (S1) with the origin of the sequences in this study and a table (S2) with the NCBI BLAST results of the newly sequenced specimens.

## Data Availability

The sequences are available at NCBI GenBank under accession numbers OP672311—OP672317 and the raw sequence data files have been deposited at BioStudies (https://www.ebi.ac.uk/biostudies/) under accession number S-BSST923.

## Abbreviations

*ASV*: Amplicon Sequence Variant
*DNA*: Deoxynucleic Acid
*GIN*: Gastrointestinal Nematode
*ITS2*: Internal Transcribed Spacer 2
*NGS*: Next Generation Sequencing
*PCR*: Polymerase Chain Reaction
*rDNA*: Ribosomal Deoxynucleic Acid
