# Supplementary material for: The complete ITS2 barcoding region for Strongylus vulgaris and Strongylus edentatus
Source: Vet Res Commun. 2023 Jan 4;47(3):1767–71. doi: 10.1007/s11259-022-10067-w (PMC10485102; doi:10.1007/s11259-022-10067-w)
Supplement: Supplementary file 1 — Supplementary file1 (PDF 292 KB) Online resource 1, contains a table (S1) with the origin of the sequences in this study and a table (S2) with the NCBI BLAST results of the newly sequenced specimens. [file 11259_2022_10067_MOESM1_ESM.pdf]

Table S1. Table with the *Strongylus* spp. sequences included in the phylogenetic tree, hosts, sample locations, accession numbers and reference publication.

| Species | Host | Location(s) | ITS2-extent[A] | Accession number(s)[B] | Reference |
|---|---|---|---|---|---|
| *Strongylus asini* | Burchell's zebra | Namibia & South Africa | Partial | X99345 | Hung et al. (1996) |
| *Strongylus edentatus* | Horse | Australia & USA | Partial | X77807 | Campbell et al. (1995) |
| *Strongylus edentatus* | Donkey | China, Henan | Partial | KP693438 | Bu et al. Unpublished |
| *Strongylus edentatus* | Horse | Canada & USA | Complete | ASV1, ASV15 | [1] Poissant et al. (2021) |
| ***Strongylus edentatus*** | **Horse** | **Sweden** | **Complete** | **OP672311 - OP672312** | **This study** |
| *Strongylus edentatus* | Horse | China, Tibet | Complete | MT193648 | Ai, et al. Unpublished |
| *Strongylus equinus* | Horse | Australia | Partial | X77808 | Campbell et al. (1995) |
| *Strongylus equinus* | Horse | Canada & USA | Complete | ASV3, ASV4, ASV26 | [1] Poissant et al. (2021) |
| *Strongylus vulgaris* | Donkey | Egypt | Partial | LC500237 | AbouLaila et al. (2020) |
| *Strongylus vulgaris* | Donkey | Egypt | Partial | OM101096, MT880779 - MT880780 | El-Gameel. et al. (2022) |
| *Strongylus vulgaris* | Equines | Iran | Partial | MT258561 - MT258562 | Alborzi et al. Unpublished |
| *Strongylus vulgaris* | Donkey | Egypt | Partial | MT090136 | El-Gameel. et al. (2022) |
| *Strongylus vulgaris* | Horse | Turkey | Partial | MF489225 - MF489226 | Yildirim and Bilgin. Unpublished |
| *Strongylus vulgaris* | Horse | Germany | Partial | KT250609 (- KT250621)[C] | Kaspar et al. (2016) |
| *Strongylus vulgaris* | Horse | Brazil, RJ | Partial | OP550136 | Correa and Barbosa. Unpublished |
| *Strongylus vulgaris* | Burchell's zebra | Kenya | Partial | OK235477 | [C] Maina et al. Unpublished |
| *Strongylus vulgaris* | Donkey | China, Henan | Partial | KP693439 | Bu et al. Unpublished |
| *Strongylus vulgaris* | Horse | Australia & USA | Partial | X77863 | Campbell et al. (1995) |
| *Strongylus vulgaris* | Horse | Canada & USA | Complete | ASV6, ASV14, ASV17, ASV18, ASV22, ASV29 | [1] Poissant et al. (2021) |
| ***Strongylus vulgaris*** | **Horse** | **Sweden** | **Complete** | **OP672313 - OP672317** | **This study** |

[A] All sequences were included in fig 2A, and only complete sequences in fig 2B.

[B] Sample sequences were downloaded from NCBI Genbank, with exception for: [1] Poissant et al. (2021), where data was extracted from raw data available at https://data.mendeley.com/datasets/vhyysw8xt2/2

[C] These sequences are identical and KT250609 was used for the phylogeny. [D] Marina et al. is available as preprint: http://dx.doi.org/10.2139/ssrn.4203693

Data searched on Oct 17, 2022.

Supplementary tables for “**The complete ITS2 barcoding region for *Strongylus vulgaris* and *Strongylus edentatus*”** by Peter Halvarsson and Eva Tydén

Table S2. BLAST search result where all sequences had a high identity to available records, even if the query cover was lower for *S. vulgaris*.

| Accession number | Species (Morphology) | Species (BLAST) | Query cover (%) | Identity (%) | Best match |
|---|---|---|---|---|---|
| OP672311 | *Strongylus edentatus* | *Strongylus edentatus* | 99 | 100 | MT193648 |
| OP672312 | *Strongylus edentatus* | *Strongylus edentatus* | 99 | 99.65 | MT193648 |
| OP672313 | *Strongylus vulgaris* | *Strongylus vulgaris* | 81 | 99.08 | MF489225 |
| OP672314 | *Strongylus vulgaris* | *Strongylus vulgaris* | 81 | 99.54 | X77863 |
| OP672315 | *Strongylus vulgaris* | *Strongylus vulgaris* | 81 | 98.62 | MF489225 |
| OP672316 | *Strongylus vulgaris* | *Strongylus vulgaris* | 81 | 99.08 | X77863 |
| OP672317 | *Strongylus vulgaris* | *Strongylus vulgaris* | 81 | 100 | MF489225 |

Reference list


AbouLaila M, Allam T, Roshdey T, Elkhatam A (2020) Strongylus vulgaris: Infection rate and molecular characterization from naturally infected donkeys at Sadat City, Egypt. Vet Parasitol Reg Stud Reports 22:100478. https://doi.org/10.1016/j.vprsr.2020.100478

Campbell AJD, Gasser RB, Chilton NB (1995) Differences in a ribosomal DNA sequence of Strongylus species allows identification of single eggs. Int J Parasitol 25:359–365. https://doi.org/10.1016/0020-7519(94)00116-6

El-Gameel SM, Al-Mokaddem AK, Salaeh NMK, Attia MM (2022) Morphomolecular characterization of Strongylus vulgaris isolated from donkeys with special references to histopathological study on the affected organs. J Parasit Dis 46:795–803. https://doi.org/10.1007/s12639-022-01498-y

Hung G-C, Jacobs DE, Krecek RC, et al (1996) Strongylus asini (Nematoda, Strongyloidea): Genetic relationships with other strongylus species determined by ribosomal DNA. Int J Parasitol 26:1407–1411. https://doi.org/10.1016/S0020-7519(96)00136-1

Kaspar A, Pfister K, Nielsen MK, et al (2016) Detection of Strongylus vulgaris in equine faecal samples by real-time PCR and larval culture – method comparison and occurrence assessment. BMC Vet Res 13:19. https://doi.org/10.1186/s12917-016-0918-y

Poissant J, Gavriliuc S, Bellaw J, et al (2021) A repeatable and quantitative DNA metabarcoding assay to characterize mixed strongyle infections in horses. Int J Parasitol 51:183–192. https://doi.org/10.1016/j.ijpara.2020.09.003
